# Machine learning identification of risk factors for heart failure in patients with diabetes mellitus with metabolic dysfunction associated steatotic liver disease (MASLD): the Silesia Diabetes-Heart Project

**DOI:** 10.1186/s12933-023-02014-z

**Published:** 2023-11-20

**Authors:** Katarzyna Nabrdalik, Hanna Kwiendacz, Krzysztof Irlik, Mirela Hendel, Karolina Drożdż, Agata M. Wijata, Jakub Nalepa, Oliwia Janota, Wiktoria Wójcik, Janusz Gumprecht, Gregory Y. H. Lip

**Affiliations:** 1 Department of Internal Medicine, Diabetology and Nephrology, Faculty of Medical Sciences in Zabrze, Medical University of Silesia, Katowice, Poland; 2 Liverpool Centre for Cardiovascular Science at University of Liverpool, Liverpool John Moores University and Liverpool Heart & Chest Hospital, Liverpool, UK; 3 Students’ Scientific Association By the Department of Internal Medicine, Diabetology and Nephrology, Faculty of Medical Sciences in Zabrze, Medical University of Silesia, Katowice, Poland; 4 Faculty of Biomedical Engineering, Silesian University of Technology, Zabrze, Poland; 5 Department of Algorithmics and Software, Silesian University of Technology, Gliwice, Poland; 6 Danish Center for Health Services Research, Department of Clinical Medicine, Aalborg University, Aalborg, Denmark

**Keywords:** Diabetes, Heart failure, Metabolic dysfunction associated steatotic liver disease, Machine learning

## Abstract

**Background:**

Diabetes mellitus (DM), heart failure (HF) and metabolic dysfunction associated steatotic liver disease (MASLD) are overlapping diseases of increasing prevalence. Because there are still high numbers of patients with HF who are undiagnosed and untreated, there is a need for improving efforts to better identify HF in patients with DM with or without MASLD. This study aims to develop machine learning (ML) models for assessing the risk of the HF occurrence in patients with DM with and without MASLD.

**Research design and methods:**

In the Silesia Diabetes-Heart Project (NCT05626413), patients with DM with and without MASLD were analyzed to identify the most important HF risk factors with the use of a ML approach. The multiple logistic regression (MLR) classifier exploiting the most discriminative patient’s parameters selected by the χ2 test following the Monte Carlo strategy was implemented. The classification capabilities of the ML models were quantified using sensitivity, specificity, and the percentage of correctly classified (CC) high- and low-risk patients.

**Results:**

We studied 2000 patients with DM (mean age 58.85 ± SD 17.37 years; 48% women). In the feature selection process, we identified 5 parameters: age, type of DM, atrial fibrillation (AF), hyperuricemia and estimated glomerular filtration rate (eGFR). In the case of MASLD( +) patients, the same criterion was met by 3 features: AF, hyperuricemia and eGFR, and for MASLD(−) patients, by 2 features: age and eGFR. Amongst all patients, sensitivity and specificity were 0.81 and 0.70, respectively, with the area under the receiver operating curve (AUC) of 0.84 (95% CI 0.82–0.86).

**Conclusion:**

A ML approach demonstrated high performance in identifying HF in patients with DM independently of their MASLD status, as well as both in patients with and without MASLD based on easy-to-obtain patient parameters.

**Graphical Abstract:**

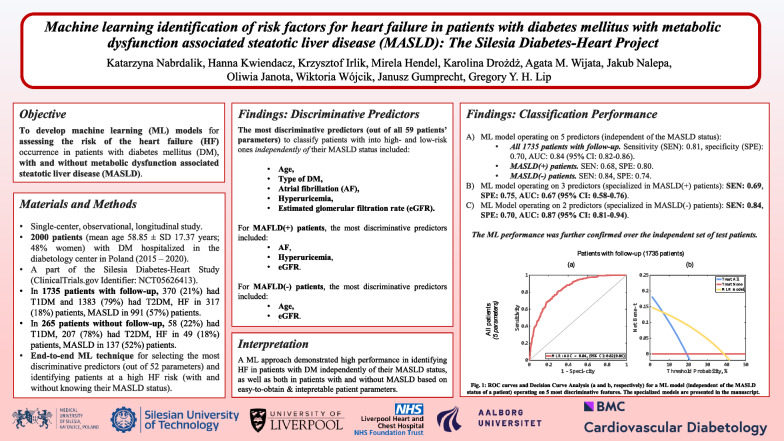

**Supplementary Information:**

The online version contains supplementary material available at 10.1186/s12933-023-02014-z.

## Background

In 2020, the concept of metabolic-associated fatty liver disease (MAFLD) has been proposed by an international expert consensus [1] to acknowledge the broader metabolic context of non-alcoholic fatty liver disease (NAFLD). The global prevalence of NAFLD has been reported to reach 30.1% [2], and prevalence of MAFLD is approx. 39% [3]. MAFLD shares many of the characteristics of NAFLD, but its definition highlights the importance of considering the entire spectrum of metabolic abnormalities, which may have a major impact on cardiovascular risk. Recognizing the link to wide spectrum of metabolic abnormalities and avoiding the stigmatizing word “fatty“, the nomenclature was recently been changed and MAFLD has now been replaced by *metabolic dysfunction associated steatotic liver disease* (MASLD) [4]. A diagnosis of MASLD is established upon meeting one of five cardiovascular risk criteria, diverging from the diagnostic approach for MAFLD, which required two out of seven metabolic dysfunctions.

Cardiovascular disease (CVD) risk is increased in patients with NAFLD which has a well-established association with metabolic syndrome [5]. Diabetes mellitus (DM) plays an essential role in MAFLD diagnosis, serving as one of the criteria for its classification and a key risk factor for its progression [6]. The international multidisciplinary consensus statement on MAFLD and the risk of CVD stresses the importance of diagnosing CVD in patients in MAFLD and assessing for MAFLD in patients with CVD [7].

In recent meta-analyses, MAFLD was also associated with left ventricular diastolic and systolic dysfunction [8, 9], strengthening the evidence for interconnection between MAFLD and CVD. Despite the high prevalence of heart failure (HF) [10], diagnosing HF presents a challenge due to its non-specific symptoms and signs in clinical practice [11]. Data on undiagnosed HF are not easy to obtain, and in up to half HF cases an accurate diagnosis may be missed [12]. Indeed, the majority of the existing tools created for predicting incident HF are based on the general population [13–15]. Within the DM population, a limited number of tools have been developed for effective HF screening, but numerous ones are devoted to prediction of incident HF [16]. Importantly, these models did not take into account MASLD as one of risk modifiers, thereby potentially limiting their utility among those affected with this condition. Given the number of patients with DM, the growing prevalence of MAFLD [17] and its impact on HF risk [8, 9], accurate approaches that can identify patients who are at a high risk of HF in patients with DM are needed, which may facilitate diagnostic accuracy and treatment planning, ultimately promoting better health outcomes.

Machine learning (ML) techniques have demonstrated potential in identifying patients at an elevated risk for incident HF [18]. We have previously reported a prospective study of patients with DM, where the multiple logistic regression (MLR) classifier was successful in identifying MAFLD patients with prevalent CVD based on easy-to-obtain parameters [19]. The current study builds upon our previous work, focusing this time on the identification of patients at high risk of HF. By selecting risk factors and creating techniques separately for patients with and without MASLD, we aimed to highlight the unique phenotypes of HF patients, which may vary based on the presence of MASLD.

In the presented study, we focused on the recently established nomenclature of MASLD, but as the terminology is new, in the manuscript we have had to refer to studies examining MAFLD given that those studying MASLD are not available as yet. The primary objective of this study was to develop and validate ML models for assessing the risk of the HF occurrence in patients with DM, amongst those with and without MASLD.

## Research design and methods

### Study design

This was an ancillary study as part of the Silesia Diabetes-Heart Project (registered on ClinicalTrials.gov with identifier NCT05626413), as previously detailed [20]. It was a single-center, observational study dedicated to examining patients with DM, who were hospitalized in the diabetology ward in Zabrze, Poland, from January 2015 to September 2020. Inclusion criteria consisted of patients aged ≥ 18 years old, with the diagnosis of either type 1 DM (T1DM) or type 2 DM (T2DM). The exclusion criteria were the terminal stages of cancer or in-hospital death. The analysis reported in this work is cross-sectional and only baseline data obtained during hospital stay were considered for MASLD and HF ascertainment.

### Ethical approval

Upon hospitalization, every patient signed consent forms, henceforth, additional consent was deemed unnecessary as the data analyzed was from an anonymized registry. This study received approval from the Medical University of Silesia Ethics Committee (PCN/0022/KB/126/20) and was conducted in accordance with the Declaration of Helsinki. Any tests and procedures employed during hospitalization were standard of medical care, and they would have been performed regardless of this study.

### Definition of heart failure

The diagnosis of HF was based on medical documentation of a previous HF diagnosis when there was sufficient evidence from medical records. Additionally, the diagnosis was made if new symptoms, signs, and structural or functional impairment of the heart was detected by echocardiography during hospitalization, based on the guidelines of the European Society of Cardiology [21]. New HF was diagnosed by an experienced consultant cardiologist who used all available diagnostic information to decide on the presence or absence of HF. Echocardiography was performed using the ARIETTA 750 ultrasound system (Hitachi) outfitted with a S121 transducer.

### MASLD diagnosis

MASLD was diagnosed through hepatic ultrasonography showing evidence of steatosis, coupled with at least one of the following criteria: T2DM or overweight or obesity (BMI ≥ 25 kg/m^2^), or blood pressure ≥ 130/85 mmHg or specific drug treatment, plasma triglycerides ≥ 1.7 mmol/L or lipid lowering treatment [4]. The ultrasonography examinations were conducted by certified radiology specialists using an ARIETTA 750 ultrasound system (Hitachi) equipped with a C253 transducer.

### Anthropometric measures, vital signs assessment and biochemical assays

Height (in meters) and weight (in kilograms) of each participant were recorded at the time of discharge using standardized methods, and their body mass index (BMI) was calculated as weight divided by the square of their height (kg/m^2^). Blood pressure and heart rate were monitored during the entire hospital stay, and the mean blood pressure and heart rate was calculated from these measurements. Blood and urine samples for biochemical tests were collected during the hospitalization period accordingly to the patient’s medical condition. An overview of biochemical methods used in this study have been described previously [20].

### Definitions of metabolic diseases

Obesity was defined based on BMI of 30 kg/m^2^ or more, and being overweight as BMI between 25 and 29.9 kg/m^2^. Diagnoses of T1DM 1and T2DM were based on prior medical history. New DM diagnoses arising during hospitalization adhered to the criteria set forth in the guidelines of the American Diabetes Association and Diabetes Poland that were operative at the time of patient’s hospitalization [22, 23]. Hypercholesterolemia was confirmed in cases where there was a documented medical history, newly discovered plasma total cholesterol levels ≥ 5 mmol/L, or if the patient was on statin therapy. Due to the lack of routine measurement of high and low-density lipoprotein during the hospital stay, it was not considered as a diagnostic parameter for hypercholesterolemia. Hypertriglyceridemia was confirmed in cases where there was a documented medical history, newly recognized plasma triglyceride levels ≥ 1.7 mmol/L, or if the patient was on fibrate therapy. Estimated glomerular filtration rate (eGFR) was calculated based on CKD-EPI (Chronic Kidney Disease Epidemiology Collaboration) equation based on the serum creatinine concentration [24]. Hyperuricemia was considered in patients with a documented medical history of the condition, or uric acid concentration that exceeded 6 mg/dl (360 µmol/L) in women and 7 mg/dl (420 µmol/L) in men.

### Definitions of cardiovascular diseases

Arterial hypertension was identified in case of a systolic blood pressure of 140 mmHg or higher, a diastolic blood pressure of 90 mmHg or higher, or treatment with antihypertensive medications. The presence of coronary artery disease was determined by either its confirmation through angiography, a history of myocardial infarction, or coronary artery bypass grafting. Atrial fibrillation (AF) was detected based on electrocardiogram (ECG) readings (12-lead ECG, 24-h ECG Holter, or other electrocardiographic documentation).

### Diabetes complications

Diabetic foot disease was confirmed by the presence of infection, ulceration, or damaged tissues of the foot. Diabetic peripheral neuropathy was diagnosed based on feet examination and symptoms of nerve dysfunction manifested by inability to sense vibration, temperature, or touch. Diabetic retinopathy was ascertained based on fundus examination during hospitalization or based on a documented medical history.

### HF risk factors in patients with DM in relation to MASLD using ML

**Fig. 1:**
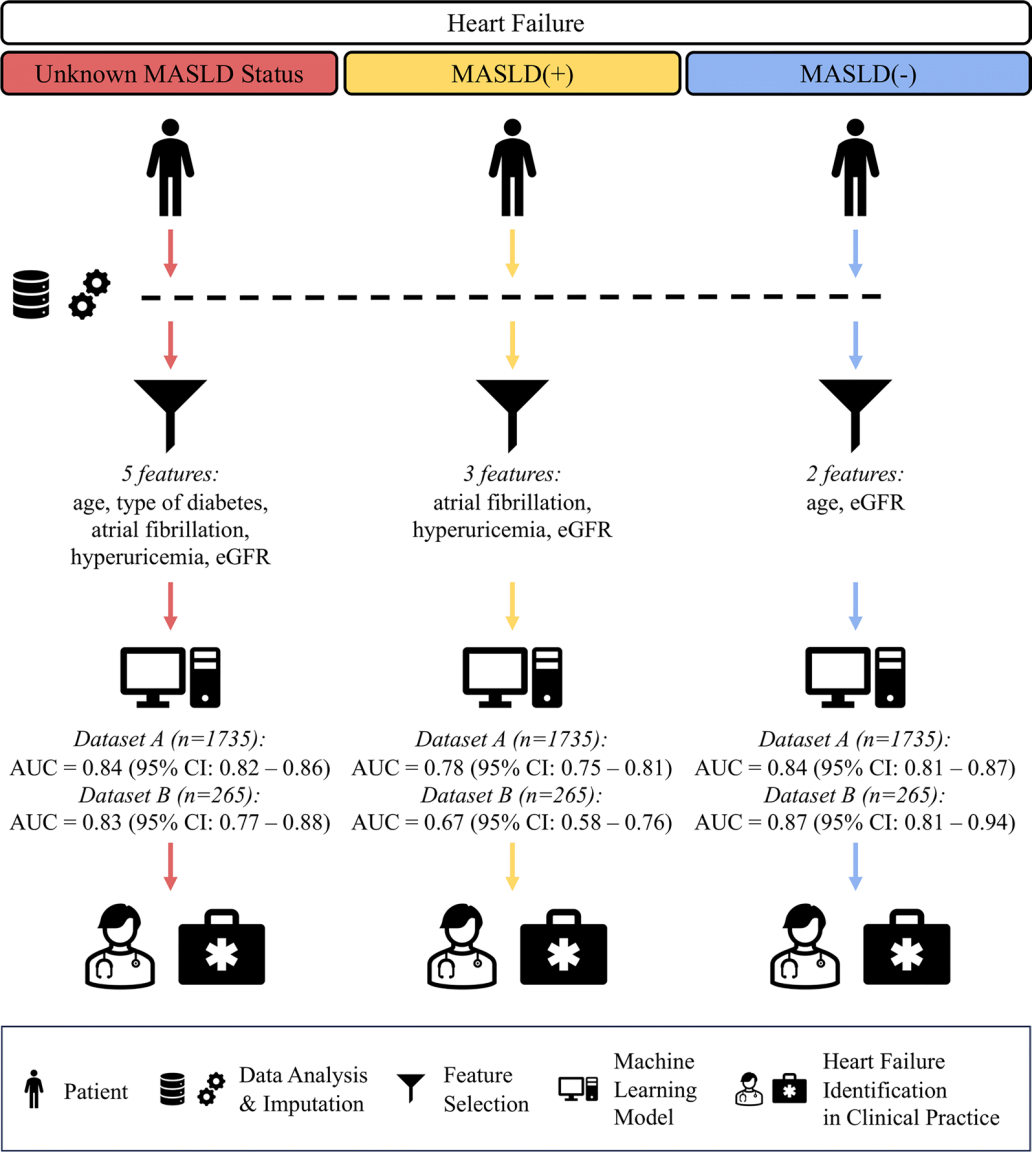
Data analysis flowchart

Identifying high risk of HF in patients with DM was performed based on the analysis of 52 parameters belonging to several categories: demographic (2 parameters), clinical (diabetes-related) (3), diabetes-related vascular disease (9), diabetic complications (3), general (3), concomitant diseases (4), and laboratory parameters (28) (Additional file 1: Table S1)—see a high-level data analysis flow of the entire pipeline in Fig. 1. Missing data were imputed using a regularized version of the factorial analysis for mixed data algorithm [25], which is based on an idea of balancing the impact of all the variables that are continuous and categorical while elaborating the dimensions of variability. The process of missing data imputation preceded the feature selection procedure and training a ML model for identifying high-risk patients.

### Determining the most discriminative patients’ parameters using feature selection

The most discriminative parameters were determined for (*i*) all patients participating in this study; for the patients (*ii*) with (MASLD( +)) and (*iii*) without [MASLD(−)] MASLD. This process was performed using the χ2 test analysis. In order to identify a stable set of the most important parameters, feature selection was repeated 1000 times following the Monte Carlo approach, with a random selection of 80% of patients, with and without HF, for each of the considered scenarios, i.e., for all patients, as well as for the MASLD( +) and for MASLD(−) subgroups (Fig. 1). The predictors for which the *p*-values determined by the χ2 test had a value lower than 0.05, being our threshold for statistical significance in this analysis, were considered significant in each iteration of the Monte Carlo analysis (those iterations are independent of each other). The final three sets of features for each scenario included only those features that were identified in all 1000 Monte Carlo iterations.

### ML classifiers for identifying patients at a high HF risk

The three sets of most discriminative parameters were used to predict HF occurrence in each of the scenarios by training MLR models, as we focus on the binary prediction outcome (a patient being either a high- or low-risk one) with predictors which may be either categorical or continuous. Also, MLR models are known to be easy to interpret yet efficient in training and reduce the effect of confounding factors [26]. For each MLR model, the cut-off value was determined based on the receiver operating characteristic curve (ROC) analysis using the Index of Union technique, which aims at maximizing both sensitivity and specificity while determining the cut-point [27]. The capabilities of the ML models were quantified using sensitivity, specificity, and the percentage of correctly classified (CC) high- and low-risk patients. We also determined the ROC curves and calculated the area under each curve (AUC) along with their 95% confidence intervals (95% CI). Decision curve analysis (DCA) was used to assess the clinical utility of the proposed solutions. In addition, we evaluated the MLR models over an independent test set of patients (i.e., those patients were never used while training ML models). Statistical analysis and ML techniques were implemented in MATLAB R2023a (feature section: fscchi2 function from the Statistics and Machine Learning Toolbox, version 2023a; MLR models: fitglm function from the Statistics and Machine Learning Toolbox, version 2023a), whereas the data imputation technique was implemented in RStudio (Build 492) using the imputeFAMD function from the missMDA package (version 1.11).

## Results

There were 2000 patients with DM (mean age 58.85 ± SD 17.37 years; 48% women) in the eligible population (Additional file 1: Figure S1).Among them we distinguished a subset of 1735 ones (Dataset A) (Additional file 1: Table S1) to select the most discriminative features and build MLR models (to ensure consistency with our previous work [20]), while the remaining 265 patients (Dataset B) –, Additional file 1: Table S2) were treated as a test set to verify the generalization of the MLR models. For all ML models, the Model Operating Points were selected using the Index of Union method (of note, although there exist other approaches toward selecting the cut-point value in the ROC analysis [28, 29], exploiting the Youden index and the closest to (0, 1) criteria led to obtaining models of similar classification performance—see Additional file 1: Table S4). In this analysis, only the baseline patient’s data was exploited.

In the Dataset A, 370 (21%) patients had T1DM and 1365 (79%) had T2DM, whereas HF was reported in 317 (18%) patients. MASLD was diagnosed in 991 (57%) patients. In the Dataset B, there were 58 (22%) with T1DM and 207 (78%) with T2DM, with HF reported in 49 (18%) patients. In this dataset, MASLD was diagnosed in 137 (52%) patients.

### Feature selection scenarios

**Fig. 2:**
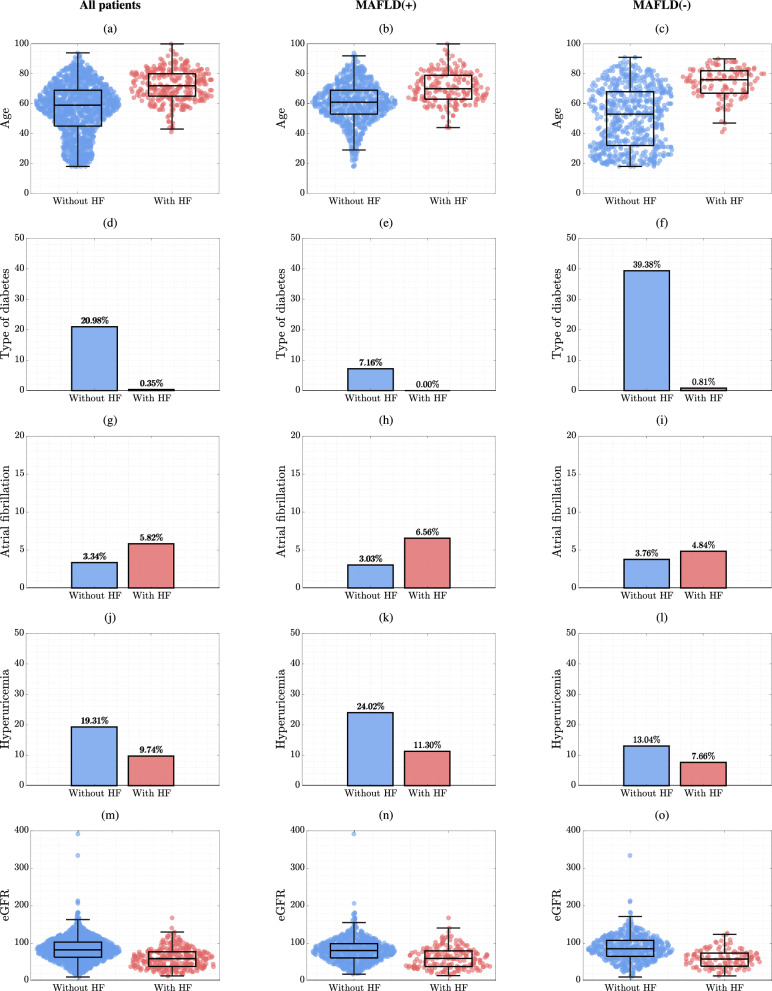
The distributions of the most discriminative features (**a**–**o**) selected for (*i*) all patients, and (*ii*) MASLD( +) and (*iii*) MASLD(−) patients only from subset of Dataset A. The bar plots present the percentages calculated with respect to the entire set of either 1735 or 265 patients (Dataset A and Dataset B, respectively)

Three feature selection scenarios resulted in 3 sets of the most discriminative predictors. For all patients from Dataset A, i.e., without knowing if they are MASLD( +) or MASLD(−), the set of selected features included 5 parameters: age, type of DM, AF, hyperuricemia and eGFR (see the values of the discriminative predictors for Dataset A in Table 1, Additional file 1: Table S1 and Fig. 2**;** and for Dataset B in Additional file 1: Table S2 and Additional file 1: Figure S2). For MASLD( +) patients, the same criterion was met by 3 features: AF, hyperuricemia and eGFR, and for MASLD(−) patients, by 2 features: age and eGFR (Fig. 2). The statistically significant differences were consistently manifested in both datasets. Of note, maintaining such small numbers of discriminative predictors is of paramount practical importance, as ML models trained over the reduced feature sets are less likely to overfit (when compared e.g., to MLR models operating over all 52 predictors), and may better generalize over different datasets [30].

**Table 1:**
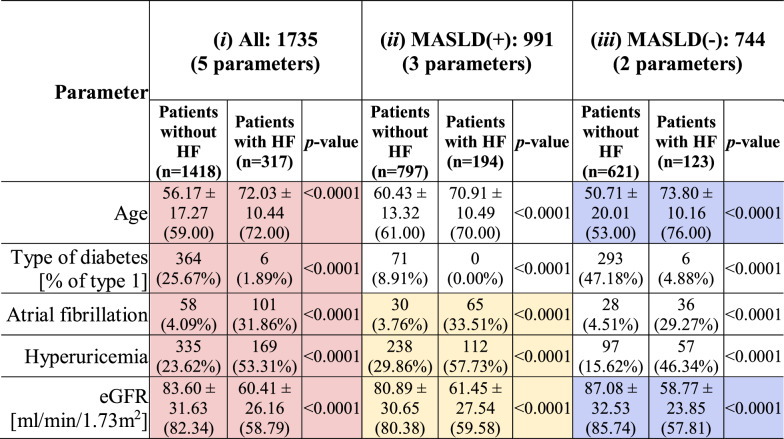
The most discriminative features (for the Dataset A) in individual scenarios (*i*) all patients: red; (*ii*) MASLD( +): blue; and (*iii*) MASLD(−) patients: yellow

The mean value ± standard deviation (SD) with the median (in parentheses) was determined for each of the analyzed continuous parameters. In the case of binary parameters, we determined the sum of 1 s and the percentage of all observations in the analyzed group. Individual comparisons of features between the groups with and without heart failure were performed by the Mann–Whitney U test (for continuous parameters) or the χ2 test (for binary)

### MLR model exploiting 5 predictors

**Fig. 3:**
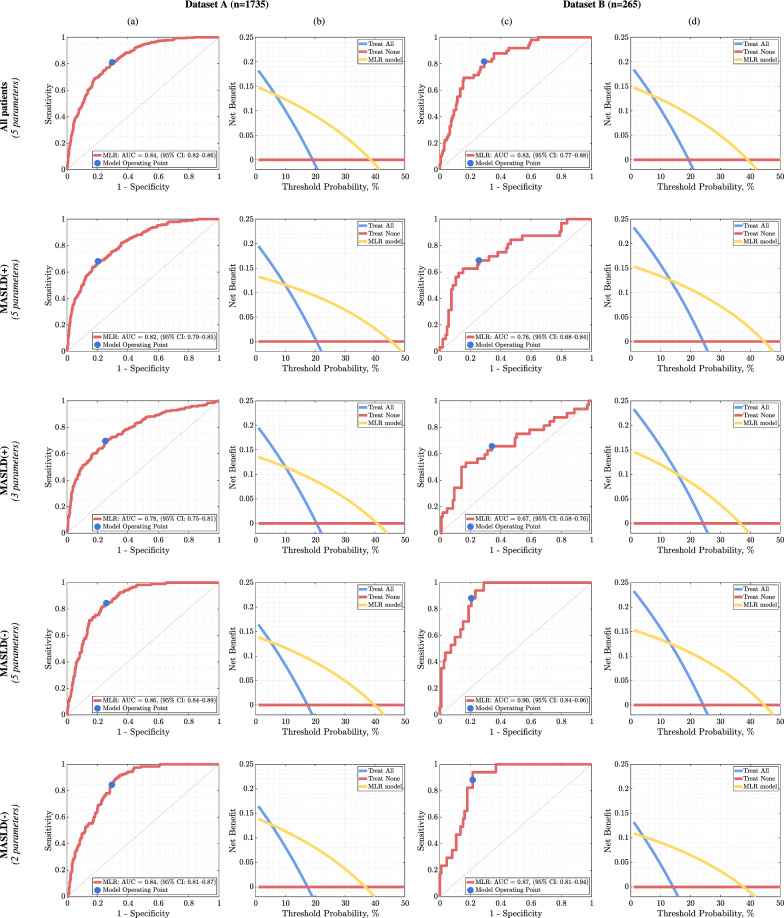
ROC curves and Decision Curve Analysis. ROC curves (**a**, **c**) obtained using the MLR models fitted to the most discriminative features determined for all patients, MASLD( +), and MASLD(−) together with the values of the areas under them. DCA curves (**b**, **d**) were estimated for each case to assess the clinical utility of the proposed classifiers. Columns a and b present results obtained for the Dataset A (1735 patients), and **c**, **d** show the results obtained for the Dataset B (265 patients)

In Dataset A, in scenario (*i*) (i.e., 5 parameters determined for all patients), 256/317 (80.76%) patients with confirmed HF, and 995/1418 (70.17%) without HF were correctly classified, hence 1251/1735 (72.10%) patients were identified correctly (Table 2). Sensitivity and specificity were 0.81 and 0.70, further confirming the ML model capabilities. Evaluation of the classifier using the ROC curves (Fig. 3a) gives AUC of 0.84 (95% CI 0.82–0.86). The analysis of clinical utility using DCA (Fig. 3b) indicates that in the range of 7% to 39% of the threshold probability, the net benefit of the MLR model based on 5 predictors was higher compared to alternative treatment strategies (treatment none and all patients).

**Table 2:** Results of predicting the occurrence of HF using three MLR models based on the most discriminative features, extracted for (*i*) all patients, (*ii*) MASLD( +) patients, and (*iii*) MASLD(−) patients of Dataset A

Method	Sensitivity	Specificity	CC with event [%]	CC without event [%]	CC All [%]
*(i)* All (5 parameters)	0.81	0.70	80.76	70.17	72.10
*(i)* MASLD( +) subgroup (5 parameters)	0.68	**0.80**	67.53	**79.55**	**77.19**
*(ii)* MASLD( +) subgroup (3 parameters)	**0.69**	0.75	**69.07**	74.65	73.56
*(i)* MASLD(−) subgroup (5 parameters)	**0.84**	**0.74**	**83.74**	**74.07**	**75.67**
*(iii)* MASLD(−) subgroup (2 parameters)	**0.84**	0.70	**83.74**	70.37	72.58

The best results in the subgroup analysis are boldfaced

### MLR model exploiting all (52) predictors

Fitting the MLR model over all (n = 52) predictors resulted in AUC: 0.96 (95% CI 0.94–0.97), with 302/317 (95.27%) and 1188/1418 (83.78%) of high- and low-risk patients correctly identified, amounting to 1490/1735 (85.88%) correctly classified patients. Testing this MLR model (fitted over all parameters) on Dataset B allowed us to correctly classify 198/265 (74.72%) patients, with 40/49 (81.63%) high- and 158/216 (73.15%) low-risk HF patients correctly identified. For this subset, AUC was 0.84 (95% CI 0.79–0.90).

Although the classification performance is consistent across the datasets, the MLR model operating on 52 patients’ parameters is much more likely to overfit when compared to our models exploiting 5, 3 or 2 most discriminative predictors (depending on the classification scenario), and may not be generalizable across different cohorts [30]. The predictive performance of the model operating on 52 patients’ parameters may be related to “memorizing” some intrinsic characteristics of the dataset—this is much less likely to happen if the number of parameters is kept small, as shown in [30]. Also, applying this model in practice is significantly more challenging, as it would require ensuring that all 52 parameters are consistently collected for the incoming patients (in contrast to up to 5 parameters utilized by our other MLR models, which could be done much faster in a day-to-day clinical practice).

### *MLR models specializing in the MASLD(* +*) and MASLD(−) subgroups*

For MASLD( +) patients (n = 991) of Dataset A, the MLR model operating on 3 parameters correctly identified 134/194 (69.07%) patients with and 595/797 (74.65%) patients without HF, resulting in 729/991 (73.56%) correct predictions. While comparing it to the 765/991 (77.19%) correctly classified MASLD( +) patients (as those with and without HF) using the MLR model based on 5 features selected in scenario (*i*), there was a similar overall classification performance of MLR using fewer features (Table 2), and the higher numbers of true positives as well false positives, i.e., the patients incorrectly identified as the high-risk ones. AUC of this model was 0.78 (95% CI 0.75–0.81) (Fig. 3a), and DCA indicates a net benefit of the proposed solution in the range of 9% to 41% of the threshold probability when compared to the all or none treatment strategies (Fig. 3b).

In the MASLD(−) subgroup analysis of Dataset A (744 patients), the MLR model was built using only two features, and correctly identified 103/123 (83.74%) patients with HF, and 437/621 (70.37%) without HF, which resulted in 540/744 (72.58%) correct predictions (Table 2). AUC of this model amounted to 0.84 (95% CI 0.81–0.87) (Fig. 3a) with a net benefit ranging from 5 to 37% compared to the all or none treatment strategies (Fig. 3b). The comparison of the MLR model operating on 2 features with the model operating on 5 features (over the same subgroup of patients) shows that the latter approach delivers better overall classification (Table 2).

### Validation of MLR models using dataset B

The MLR models for the three considered scenarios were evaluated using an independent Dataset B (n = 265; see the feature values in Additional file 1: Table S3). Here, 192/265 (72.45%) patients were correctly classified using 5 features (AUC of 0.83, 95% CI 0.77–0.88 with the clinical benefit of 7%-39% according to DCA), with 39/49 (79.59%) and 153/216 (70.83%) MASLD( +) and MASLD(−) patients correctly identified as those with high HF risk (Additional file 1: Table S5). These results are consistent with those obtained for Dataset A (n = 1735) (Table 2). Subgroup evaluation of the models showed that in scenario (*ii*), where a specialized ML model for MASLD( +) patients operating over 3 features was built (AUC of 0.67, 95% CI 0.58–0.76 with the clinical benefit of 13%-45% according to DCA), the model tended to classify more healthy patients as those with high HF risk (leading to false positives), when compared to the 5-feature model. In the MASLD(−) subgroup, there were no differences between the models based on (*i*) 5 and (*iii*) 2 features, suggesting that the model requiring less predictors should be used—AUC was 0.87 (95% CI 0.81–0.94), while the clinical benefit ranged from 4 to 38% according to DCA (Fig. 3c, d).

## Discussion

In this analysis, our principal findings are as follows: (1) HF in patients with DM can be effectively identified with the use of a small subset of the most discriminative parameters exploited to build a MLR model; (2) in general population of patients with DM regardless of their MASLD status, utilizing 5 clinical parameters which are easy-to-obtain in clinical practice (age, type of DM, AF, hyperuricemia, and eGFR) was enough to identify patients who have concomitant HF; (3) in patients with DM and comorbid MASLD, using only three parameters (AF, hyperuricemia, and eGFR) was enough to identify patients who present with HF; and (4) in patients with DM without comorbid MASLD, using only two clinical parameters (age and eGFR) allowed to identify patients who have HF.

Several acknowledged risk factors for incident HF in DM include older age, longer duration of DM, cumulative glycemic burden, higher BMI, atherosclerotic disease, elevated urinary albumin concentration, impaired renal function and hypertension [33, 34]. However, these risk factors cannot provide more personalized information about the risk of HF in a particular patient with DM because—for a clinician—it would be essential to know the exact constellation of parameters which indicate the high probability that a patient who has just been referred for the first time is at high risk of HF and should be diagnosed with this disease. Our study suggests that this answer could be obtained from the ML models. In general population of patients with DM independently of the MASLD status, the model operating on 5 features achieved high predictive performance in identifying patients with HF.

Our findings provide practical and easy to implement information about the risk factors of HF in patients with DM. Specifically, eGFR is negatively associated with the risk of HF, and others are positively associated with the HF risk, i.e., T2DM rather than T1DM, age, AF, and hyperuricemia. Several predictive models have surfaced in recent years that aimed to assess the risk of incident HF in individuals with DM, utilizing a range of factors. Williams et al. drew from electronic medical records to pinpoint predictors of HF hospitalization, including age, coronary artery disease, blood urea nitrogen, AF, and hemoglobin A1c (HbA1c), among others [33]. Meanwhile, Hippisley-Cox’s QRISK score discerned a series of parameters including systolic blood pressure, ethnicity, DM duration, type of DM and AF [34]. A post-hoc analysis of the PROactive study formulated a risk score, highlighting predictors such as age, elevated serum creatinine, diuretic use, HbA1c, duration of DM, low-density lipoprotein cholesterol, heart rate, right and left bundle branch block, microalbuminuria, previous myocardial infarction, and pioglitazone treatment [35]. Another study derived DM-CURE risk score, based on the data from the Action to Control Cardiovascular Risk in Diabetes (ACCORD) trial. It spotlighted age at T2DM diagnosis, healthcare utilization, and cardiovascular-related variables as most substantial predictors [36]. In another analysis of ACCORD trial, Segar et al. [18] developed a ML-derived (random survival forest) risk score which—similarly to us—used readily available clinical, laboratory, and additionally electrocardiographic variables. Eventually, the process of feature selection resulted in inclusion of BMI, age, hypertension, creatinine, high-density lipoprotein cholesterol, fasting plasma glucose, QRS duration, among others, as optimal predictors. Our predictive model for DM patients echoes many of these elements, including reflection of renal function by eGFR, but further introduces uric acid levels. The above mentioned models, however, were different from ours which aimed to show that the patient has the HF at the time of examination, not for predicting the future.

The subgroup analysis of patients, either with or without MASLD, revealed two distinct smaller sets of discriminative features which were subsets of 5 most important predictors elaborated for the entire cohort. This substantiates the premise that the MASLD status essentially splits patients with HF into unique phenotypes, setting a basis for specialized models that can operate on reduced number of features. These models are finely tuned for specific groups of patients delineated based on their MASLD status, and intrinsically gain from this added layer of clinical data, without the loss of classification performance. Practically speaking, this allows for a simplified approach to HF risk assessment, using fewer factors but still maintaining a similar level of accuracy. To draw a parallel, crafting specialized models for patients with known MASLD status, could be compared to the derivation of specialized models for males and females. This approach allows for selection of most predictive features that are sex-specific [37].

For the model developed specifically for patients with MASLD, only three clinical features were deemed most predictive of HF: presence of AF, hyperuricemia and reduced eGFR. All of them are associated with metabolic syndrome and both hyperuricemia and eGFR are indicative of renal dysfunction. Among the phenotype with MASLD, the cardiometabolic multimorbidity makes the presence of HF much more probable, beyond just one’s age. Interestingly, the irrelevance of type of DM likely stems from low rate of HF among those with T1DM. On the other hand, the model created only for those unaffected by MASLD demonstrates only older age and diminished eGFR as most useful to identify HF. The role of age stands pivotal, and it appears to be more telling than other clinical markers in identifying the HF. This finding is the reflection of the composite physiological alterations and their consequential impact on cardiac health, thereby serving as the main indicator for HF screening in individuals without MASLD. Supporting this observation, pooled population-based cohort study revealed that although incidence of HF substantially rises with age, and there is a significant interaction between age and established risk factors for HF such as DM, myocardial infarction, and AF. These risk factors conferred a greater risk for incident HF with either reduced or preserved ejection fraction in young compared to elderly participants. Consequently, these risk factors present a lower population attributable risk among the elderly [38].

Once again, as MASLD is a novel terminology, we have to compare our results to analysis of MAFLD and NAFLD. In the meta-analysis by Alon et al. there was a suggestion of an association of NAFLD with an increased risk of HF, AF, myocardial infarction and ischemic stroke [40] but later one by Zhou et al. highlighted a current lack of sufficient evidence to establish an association between MAFLD and AF [39]. In our study on the other hand, we indicated AF as one of the three factors enabling to detect HF among MASLD patients. Of note, eGFR was selected as a predictor in all of the abovementioned scenarios (i.e., the unknown MASLD status, and the patients with or without MASLD). These observations highlight the universality of eGFR as a marker of HF. Similarly, The Atherosclerosis Risk in Communities Study demonstrated that reduced eGFR increases the risk of incident HF among those both with and without a history of coronary heart disease [41].

Most MAFLD patients have co-existing obesity, however, MAFLD is also common in population without obesity and—among these patients—there is a higher risk of developing CVD [42]. It has been proven that non-obese MAFLD patients and patients with MAFLD and DM had a higher risk of mortality [43]. Therefore, to improve risk assessment for MASLD patients, it is important to classify subgroups within the MASLD population based on metabolic phenotypes that consider the presence of metabolic disorders. Building on this need, we focused our study on the distinctive population with both DM and MASLD. This approach could be instrumental in determining the varying levels of risk among patients with MASLD, especially when there are no approved pharmaceutical treatments and lifestyle changes remain the main MASLD treatment option that is recommended [44, 45]. Moreover, to improve both individualized management and overall public health outcomes in the context of MAFLD, two-step screening strategy combining BMI and lipid accumulation product index has been recently proposed [46].

Validation of the models proved that they discriminated the subset of patients in Dataset B with broadly similar accuracy when compared to Dataset A, showing that the models did not overfit and are able to generalize well over the unseen data (overall, the classification capabilities of the MLR models were verified over 2000 patients in total). For the scenario operating on 3 features in MASLD(+) patients and on 2 features in MASLD(−) patients, the model tended to classify more healthy patients as those having HF, while in fact they do not (hence, leading to false-positive detections). Fortunately, the predictive model for MASLD(+) individuals exhibited slightly higher accuracy in correctly classifying patients with HF. Even though there are visible differences in cohorts with and without follow-up, the model could identify patients who had HF which confirms its generalizability.

Summarizing, we automatically selected the most discriminative features from an extensive range of clinical and laboratory parameters, with the aim to enhance the precision of HF risk evaluation in patients with and without MASLD using specialized ML models. Second, we introduced a model which could identify patients with a HF risk independently of their MASLD status. This approach opens new doors toward building cascaded classification systems combining the identification of patients with MASLD and assessing their HF risk in a reproducible way. Future studies should also incorporate physical activity as one of the parameters that could modify patients prognosis since exercise increases myocardial free fatty acid oxidation in subjects with MAFLD what can be important in term of HF [47].

### Limitations

We are aware of several limitations of our study. This was a single center study what may limit its generalizability. We did not collect the information about the cases of gout what could be important—e.g., as additional predictors in identifying high-risk patients. We also did not analyze patients’ blood lactate levels which in a recent real-world study turned out to be associated with an increased risk of MAFLD in patients with T2DM [48]. In our diagnosis of new onset HF, we were limited by the absence of natriuretic peptide measurements, vital in cases of HF with preserved ejection fraction. Additionally, we were not able to phenotype HF based on ejection fraction, due to the lack of echocardiography data for some of the patients. Finally, although MLR models offer high-quality classification and generalized over unseen patients’ data, deploying other well-established ML techniques [49] also including deep ML classifiers [50] could further improve the classification performance of the proposed pipeline.

## Conclusion

Older age, T2DM, AF, hyperuricemia and lower eGFR discriminate patients who are at a high HF risk. A ML approach demonstrated high performance in identifying HF in patients with DM, as well as both in patients with and without MASLD based on easy-to-obtain parameters.

### Supplementary Information

**Additional file 1: Figure S1.** Study flowchart. **Figure S2.** Distribution of most discriminative parameters. **Table S1.** Baseline characteristic of participants in Dataset A. **Table S2.** Baseline characteristic of participants in Dataset B. **Table S3.** The most discriminative features for the patients in Dataset B. **Table S4.** The ROC analysis for extracting cut-points. **Table S5.** Performance metrics of the model in in Dataset B.

## Data Availability

The datasets generated during and analyzed in the current study are available from the corresponding author upon reasonable request.

## Abbreviations

ACCORD: Action to control cardiovascular risk in diabetes
AF: Atrial fibrillation
AUC: Area under the curve
BMI: Body mass index
CC: Correctly classified
CKD-EPI: Chronic kidney disease epidemiology collaboration
CI: Confidence interval
CVD: Cardiovascular disease
DCA: Decision curve analysis
DM: Diabetes mellitus
ECG: Electrocardiogram
eGFR: Estimated glomerular filtration rate
HF: Heart failure
MAFLD: Metabolic dysfunction-associated fatty liver disease
MASLD: Metabolic dysfunction-associated steatotic liver disease
ML: Machine learning
MLR: Multiple logistic regression
ROC: Receiver operating characteristic
T1DM: Type 1 diabetes mellitus
T2DM: Type 2 diabetes mellitus

## References

[1] Eslam M, Newsome PN, Sarin SK, Anstee QM, Targher G, Romero-Gomez M (2020). A new definition for metabolic dysfunction-associated fatty liver disease: an international expert consensus statement. J Hepatol.

[2] Younossi ZM, Golabi P, Paik JM, Henry A, Van Dongen C, Henry L (2023). The global epidemiology of nonalcoholic fatty liver disease (NAFLD) and nonalcoholic steatohepatitis (NASH): a systematic review. Hepatology.

[3] Lim GEH, Tang A, Ng CH, Chin YH, Lim WH, Tan DJH (2023). An observational data meta-analysis on the differences in prevalence and risk factors between MAFLD vs NAFLD. Clin Gastroenterol Hepatol.

[4] Rinella ME, Lazarus JV, Ratziu V, Francque SM, Sanyal AJ, Kanwal F (2023). A multi-society Delphi consensus statement on new fatty liver disease nomenclature. Hepatology.

[5] Tana C, Ballestri S, Ricci F, Di Vincenzo A, Ticinesi A, Gallina S (2019). Cardiovascular risk in non-alcoholic fatty liver disease: mechanisms and therapeutic implications. Int J Environ Res Public Health.

[6] Eslam M, Sanyal AJ, George J, International Consensus Panel (2020). MAFLD: A Consensus-Driven Proposed Nomenclature for Metabolic Associated Fatty Liver Disease. Gastroenterology..

[7] Zhou X-D, Targher G, Byrne CD, Somers V, Kim SU, Chahal CAA (2023). An international multidisciplinary consensus statement on MAFLD and the risk of CVD. Hepatol Int.

[8] Yong JN, Ng CH, Lee CW-M, Chan YY, Tang ASP, Teng M (2022). Non-alcoholic fatty liver disease association with structural heart, systolic and diastolic dysfunction: a meta-analysis. Hepatol Int.

[9] Borges-Canha M, Neves JS, Libânio D, Von-Hafe M, Vale C, Araújo-Martins M (2019). Association between nonalcoholic fatty liver disease and cardiac function and structure-a meta-analysis. Endocrine.

[10] Savarese G, Lund LH (2017). global public health burden of heart failure. Card Fail Rev.

[11] Arroll B, Doughty R, Andersen V (2010). Investigation and management of congestive heart failure. BMJ.

[12] Barents M, van der Horst ICC, Voors AA, Hillege JL, Muskiet FA, de Jongste MJ (2008). Prevalence and misdiagnosis of chronic heart failure in nursing home residents: the role of B-type natriuretic peptides. Neth Heart J.

[13] Khan SS, Ning H, Shah SJ, Yancy CW, Carnethon M, Berry JD (2019). 10-Year risk equations for incident heart failure in the general population. J Am Coll Cardiol.

[14] Agarwal SK, Chambless LE, Ballantyne CM, Astor B, Bertoni AG, Chang PP (2012). Prediction of incident heart failure in general practice. Circul Heart Fail.

[15] Chahal H, Bluemke DA, Wu CO, McClelland R, Liu K, Shea SJ (2015). Heart failure risk prediction in the multi-ethnic study of atherosclerosis. Heart.

[16] Boonman-de Winter LJM, Rutten FH, Cramer MJ, Landman MJ, Zuithoff NPA, Liem AH (2015). Efficiently screening heart failure in patients with type 2 diabetes. Eur J Heart Fail.

[17] Wong W-K, Chan W-K (2021). Nonalcoholic fatty liver disease: a global perspective. Clin Ther.

[18] Segar MW, Vaduganathan M, Patel KV, McGuire DK, Butler J, Fonarow GC (2019). Machine learning to predict the risk of incident heart failure hospitalization among patients with diabetes: the watch-dm risk score. Diabetes Care.

[19] Drożdż K, Nabrdalik K, Kwiendacz H, Hendel M, Olejarz A, Tomasik A (2022). Risk factors for cardiovascular disease in patients with metabolic-associated fatty liver disease: a machine learning approach. Cardiovasc Diabetol.

[20] Nabrdalik K, Kwiendacz H, Drożdż K, Irlik K, Hendel M, Wijata AM (2023). Machine learning predicts cardiovascular events in patients with diabetes: the silesia diabetes-heart project. Curr Probl Cardiol.

[21] McDonagh TA, Metra M, Adamo M, Gardner RS, Baumbach A, Böhm M (2021). 2021 ESC Guidelines for the diagnosis and treatment of acute and chronic heart failure: developed by the task force for the diagnosis and treatment of acute and chronic heart failure of the European society of cardiology (ESC) With the special contribution of the heart failure association (HFA) of the ESC. Eur Heart J.

[22] ElSayed NA, Aleppo G, Aroda VR, Bannuru RR, Brown FM, Bruemmer D (2022). Classification and diagnosis of diabetes: standards of care in diabetes—2023. Diabetes Care.

[23] Araszkiewicz A, Bandurska-Stankiewicz E, Borys S, Budzyński A, Cyganek K, Cypryk K (2023). 2023 Guidelines on the management of patients with diabetes—a position of diabetes Poland. Current Topics in Diabetes.

[24] Inker LA, Schmid CH, Tighiouart H, Eckfeldt JH, Feldman HI, Greene T (2012). Estimating glomerular filtration rate from serum creatinine and cystatin C. N Engl J Med.

[25] Audigier V, Husson F, Josse J (2016). A principal component method to impute missing values for mixed data. Adv Data Anal Classif.

[26] Sperandei S (2014). Understanding logistic regression analysis. Biochem Med.

[27] Unal I (2017). Defining an optimal cut-point value in ROC analysis: an alternative approach. Comput Math Methods Med.

[28] Hosseini Mojahed F, Aalami AH, Pouresmaeil V, Amirabadi A, Qasemi Rad M, Sahebkar A (2020). Clinical evaluation of the diagnostic role of microrna-155 in breast cancer. Int J Genomics.

[29] Santulli G, Pascale V, Finelli R, Visco V, Giannotti R, Massari A (2019). We are what we eat: impact of food from short supply chain on metabolic syndrome. J Clin Med.

[30] Lever J, Krzywinski M, Altman N (2016). Model selection and overfitting. Nat Methods.

[31] Vaur L, Gueret P, Lievre M, Chabaud S, Passa P, DIABHYCAR Study Group (type 2 DIABetes, Hypertension, Cardiovascular Events and Ramipril) study (2003). Development of congestive heart failure in type 2 diabetic patients with microalbuminuria or proteinuria: observations from the DIABHYCAR (type 2 diabetes, hypertension, cardiovascular events and ramipril) study. Diabetes Care.

[32] Nichols GA, Gullion CM, Koro CE, Ephross SA, Brown JB (2004). The incidence of congestive heart failure in type 2 diabetes: an update. Diabetes Care.

[33] Williams BA, Geba D, Cordova JM, Shetty SS (2020). A risk prediction model for heart failure hospitalization in type 2 diabetes mellitus. Clin Cardiol.

[34] Hippisley-Cox J, Coupland C (2015). Development and validation of risk prediction equations to estimate future risk of heart failure in patients with diabetes: a prospective cohort study. BMJ Open.

[35] Pfister R, Cairns R, Erdmann E, Schneider CA (2013). A clinical risk score for heart failure in patients with type 2 diabetes and macrovascular disease: an analysis of the PROactive study. Int J Cardiol.

[36] Lin Y, Shao H, Shi L, Anderson AH, Fonseca V (2022). Predicting incident heart failure among patients with type 2 diabetes mellitus: the DM-CURE risk score. Diabetes Obes Metab.

[37] Sun LY, Zghebi SS, Eddeen AB, Liu PP, Lee DS, Tu K (2022). Derivation and external validation of a clinical model to predict heart failure onset in patients with incident diabetes. Diabetes Care.

[38] Tromp J, Paniagua SMA, Lau ES, Allen NB, Blaha MJ, Gansevoort RT (2021). Age dependent associations of risk factors with heart failure: pooled population based cohort study. BMJ.

[39] Zhou B-G, Ju S-Y, Mei Y-Z, Jiang X, Wang M, Zheng A-J (2023). A systematic review and meta-analysis of cohort studies on the potential association between NAFLD/MAFLD and risk of incident atrial fibrillation. Front Endocrinol.

[40] Alon L, Corica B, Raparelli V, Cangemi R, Basili S, Proietti M (2022). Risk of cardiovascular events in patients with non-alcoholic fatty liver disease: a systematic review and meta-analysis. Eur J Prev Cardiol.

[41] Kottgen A, Russell SD, Loehr LR, Crainiceanu CM, Rosamond WD, Chang PP (2007). Reduced kidney function as a risk factor for incident heart failure: the atherosclerosis risk in communities (ARIC) study. J Am Soc Nephrol.

[42] Huang Z, Ye D, Loomes K, Cheng KK, Hui HX (2023). Editorial: Metabolic associated fatty liver disease: clinical perspectives from pathogenesis to diagnosis and treatment. Front Endocrinol.

[43] Chung GE, Yu SJ, Yoo J-J, Cho Y, Lee K, Shin DW (2023). Lean or diabetic subtypes predict increased all-cause and disease-specific mortality in metabolic-associated fatty liver disease. BMC Med.

[44] Rinella ME, Neuschwander-Tetri BA, Siddiqui MS, Abdelmalek MF, Caldwell S, Barb D (2023). AASLD Practice guidance on the clinical assessment and management of nonalcoholic fatty liver disease. Hepatology.

[45] Chai X-N, Zhou B-Q, Ning N, Pan T, Xu F, He S-H (2023). Effects of lifestyle intervention on adults with metabolic associated fatty liver disease: a systematic review and meta-analysis. Front Endocrinol.

[46] Wang H, Zhang Y, Liu Y, Li H, Xu R, Fu H (2023). Comparison between traditional and new obesity measurement index for screening metabolic associated fatty liver disease. Front Endocrinol.

[47] Risikesan J, Heebøll S, Kumarathas I, Funck KL, Søndergaard E, Johansen RF (2023). Exercise increases myocardial free fatty acid oxidation in subjects with metabolic dysfunction-associated fatty liver disease. Atherosclerosis.

[48] Ma Y-L, Ke J-F, Wang J-W, Wang Y-J, Xu M-R, Li L-X (2023). Blood lactate levels are associated with an increased risk of metabolic dysfunction-associated fatty liver disease in type 2 diabetes: a real-world study. Front Endocrinol.

[49] Nalepa J, Kawulok M (2019). Selecting training sets for support vector machines: a review. Artif Intell Rev.

[50] Sarker IH (2021). Deep learning: a comprehensive overview on techniques, taxonomy, applications and research directions. SN Comput Sci.

