# Supplementary material for: Machine learning identification of risk factors for heart failure in patients with diabetes mellitus with metabolic dysfunction associated steatotic liver disease (MASLD): the Silesia Diabetes-Heart Project
Source: Cardiovasc Diabetol. 2023 Nov 20;22:318. doi: 10.1186/s12933-023-02014-z (PMC10661663; doi:10.1186/s12933-023-02014-z)
Supplement: Supplementary file 1 — Additional file 1: Figure S1. Study flowchart. Figure S2. Distribution of most discriminative parameters. Table S1. Baseline characteristic of participants in Dataset A. Table S2. Baseline characteristic of participants in Dataset B. Table S3. The most discriminative features for the patients in Dataset B. Table S4. The ROC analysis for extracting cut-points. Table S5. Performance metrics of the model in in Dataset B. [file 12933_2023_2014_MOESM1_ESM.docx]

**SUPPLEMENTARY MATERIAL**


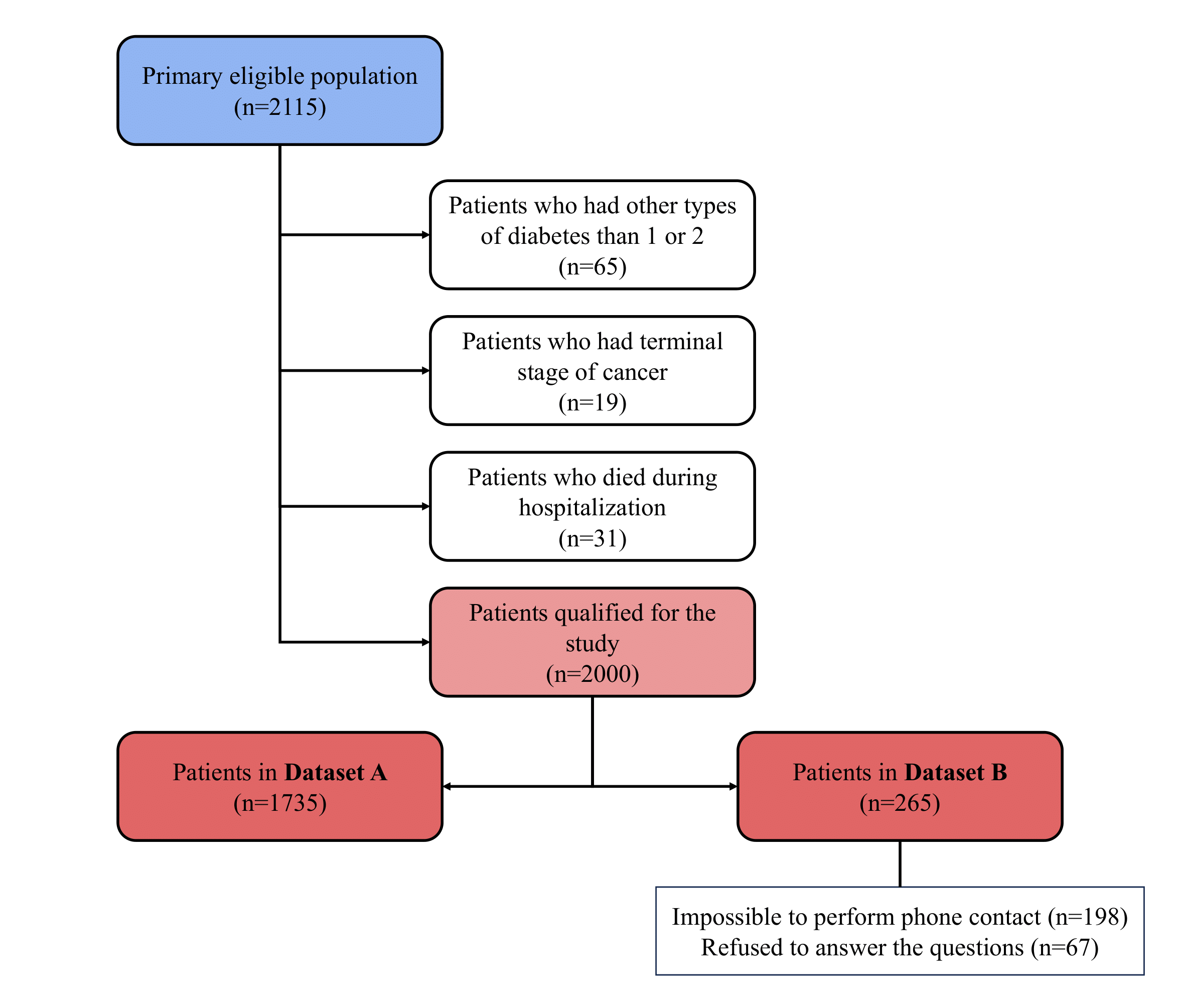


**Supplementary Figure 1:** Patient flowchart.


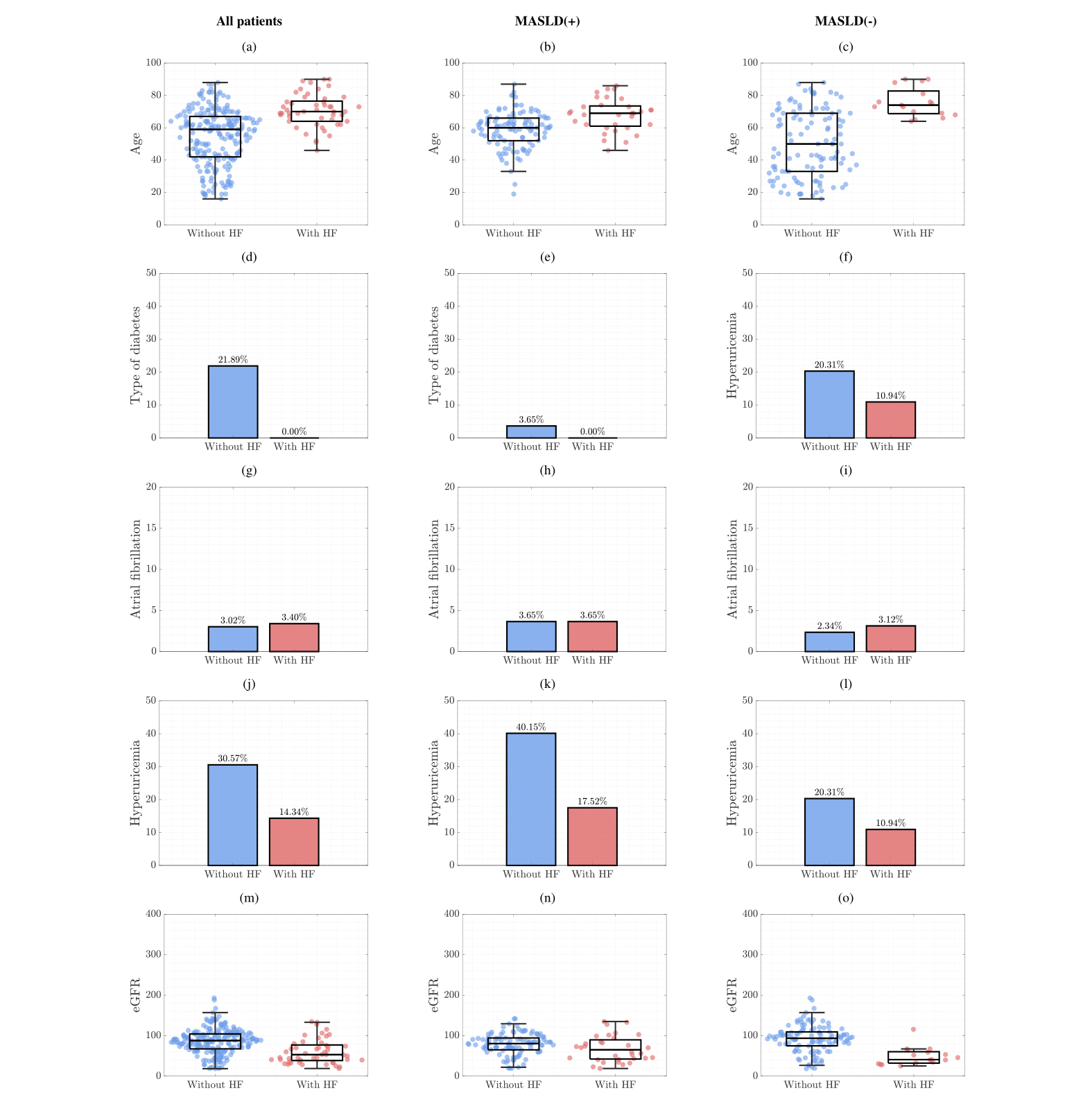
**Supplementary Figure 2:** The distributions of the most discriminative features (a-o) selected for (*i*) all patients, and (*ii*) MASLD(+) and (*iii*) MASLD(-) patients only from subset of Dataset B.

**Supplementary Table 1**: Clinical patient parameters of Dataset A.

The most discriminative features for all patients are bold and red.

| **Parameter** | **Patients without HF**  **(n=1418)** | **Patients with HF**  **(n=317)** | ***p*-value** |
| --- | --- | --- | --- |
| ***Demographic parameters*** | | | |
| **Age** | **56.17 ± 17.27 (59.00)** | **72.03 ± 10.44 (72.00)** | **<0.0001** |
| Men, n (%) | 675 (47.60%) | 0.44 ± 0.50 (0.00) | 0.314 |
| ***Clinical parameters*** | | | |
| *Diabetes-related* | | | |
| BMI [kg/m^2^] | 29.71 ± 6.82 (29.40) | 32.57 ± 6.51 (31.83) | <0.0001 |
| Duration of diabetes [years] | 11.07 ± 8.64 (10.00) | 13.35 ± 7.75 (13.41) | <0.0001 |
| **Type of diabetes [% of type 1]** | **364 (25.67%)** | **6 (1.89%)** | **<0.0001** |
| *Cardiovascular-related* | | | |
| **Atrial fibrillation** | **58 (4.09%)** | **101 (31.86%)** | **<0.0001** |
| Carotid arteries stenosis | 21 (1.48%) | 8 (2.52%) | 0.190 |
| Coronary artery disease | 300 (21.16%) | 313 (98.74%) | <0.0001 |
| Hypertension | 974 (68.69%) | 292 (92.11%) | <0.0001 |
| Mean diastolic blood pressure [mmHg] | 76.52 ± 7.28 (77.00) | 75.28 ± 7.94 (76.00) | 0.018 |
| Mean heart rate [bpm] | 79.39 ± 13.95 (80.00) | 84.60 ± 18.33 (80.00) | <0.001 |
| Mean systolic blood pressure [mmHg] | 128.02 ± 14.64 (127.00) | 128.29 ± 15.51 (128.00) | 0.602 |
| Peripheral artery disease | 54 (3.81%) | 27 (8.52%) | 0.000 |
| Stroke | 110 (7.76%) | 34 (10.73%) | 0.083 |
| *Diabetic complications* | | | |
| Diabetic foot disease | 38 (2.68%) | 12 (3.79%) | 0.287 |
| Diabetic peripheral neuropathy | 133 (9.38%) | 19 (5.99%) | 0.054 |
| Retinopathy | 525 (37.02%) | 103 (32.49%) | 0.129 |
| *General* | | | |
| Current smoker [% of yes] | 265 (18.69%) | 53 (16.72%) | 0.413 |
| Emergency admission [% of yes] | 331 (23.34%) | 134 (42.27%) | <0.0001 |
| Number of days of hospital stay | 7.08 ± 2.64 (7.00) | 8.16 ± 3.32 (8.00) | <0.0001 |
| *Concomitant diseases* | | | |
| Degenerative disease of the spine | 517 (36.46%) | 138 (43.53%) | 0.019 |
| Hypercholesterolemia | 919 (64.81%) | 194 (61.20%) | 0.226 |
| Hypertriglyceridemia | 539 (38.01%) | 112 (35.33%) | 0.373 |
| **Hyperuricemia** | **335 (23.62%)** | **169 (53.31%)** | **<0.0001** |
| **Laboratory parameters** | | | |
| Alanine aminotransaminase [U/L] | 33.01 ± 37.16 (23.30) | 37.02 ± 132.24 (22.10) | 0.023 |
| Aspartate aminotransaminase [U/L] | 31.37 ± 45.42 (22.35) | 40.50 ± 171.06 (22.90) | 0.324 |
| Basophil count [10^9^/L] | 0.04 ± 0.05 (0.02) | 0.04 ± 0.09 (0.03) | 0.805 |
| CRP [mg/L] | 17.02 ± 50.54 (3.15) | 30.96 ± 60.90 (6.72) | <0.0001 |
| **eGFR [ml/min/1.73m^2^]** | **83.60 ± 31.63 (82.34)** | **60.41 ± 26.16 (58.79)** | **<0.0001** |
| Eosinophil count [10^9^/L] | 0.19 ± 0.46 (0.15) | 0.20 ± 0.17 (0.15) | 0.074 |
| HbA1c [%] | 9.21 ± 2.28 (8.88) | 8.38 ± 2.17 (7.94) | <0.0001 |
| HCT [%] | 40.16 ± 5.82 (40.70) | 38.84 ± 6.18 (39.60) | <0.0001 |
| Hgb [g/dL] | 13.70 ± 2.11 (13.90) | 13.04 ± 2.25 (13.20) | <0.0001 |
| Ketones – urine sample | 321 (22.64%) | 34 (10.73%) | <0.0001 |
| Lymphocyte count [10^9^/L] | 2.17 ± 1.60 (2.07) | 2.65 ± 6.43 (1.91) | 0.011 |
| MCH [pg] | 30.72 ± 2.80 (30.60) | 30.50 ± 2.76 (30.40) | 0.109 |
| MCHC [g/dL] | 34.02 ± 1.28 (34.10) | 33.52 ± 1.30 (33.50) | <0.0001 |
| MCV [fL] | 90.25 ± 6.72 (90.00) | 90.72 ± 6.65 (90.30) | 0.130 |
| Mean fast. Glycemia [mg/dL] first day | 198.38 ± 83.11 (183.50) | 183.61 ± 78.12 (170.00) | <0.001 |
| Mean fast. Glycemia [mg/dL] last day | 135.43 ± 35.77 (132.00) | 137.96 ± 36.84 (135.45) | 0.340 |
| Mean post. Glycemia [mg/dL] first day | 177.02 ± 67.21 (166.00) | 173.82 ± 58.29 (162.00) | 0.374 |
| Mean post. Glycemia [mg/dL] last day | 139.06 ± 29.66 (136.00) | 141.07 ± 30.58 (136.00) | 0.375 |
| Monocyte count [10^9^/L] | 0.62 ± 0.36 (0.54) | 0.79 ± 0.94 (0.63) | <0.0001 |
| Neutrophil count [10^9^/L] | 5.65 ± 3.58 (4.82) | 7.25 ± 5.21 (5.84) | <0.0001 |
| Platelet count [10^9^/L] | 248.09 ± 87.34 (241.00) | 254.52 ± 110.84 (232.00) | 0.694 |
| Potassium [mmol/L] | 4.58 ± 0.55 (4.55) | 4.69 ± 0.66 (4.65) | 0.003 |
| Protein – urine sample | 544 (38.36%) | 174 (54.89%) | <0.0001 |
| Red blood cell count [10^12^/L] | 4.48 ± 0.68 (4.54) | 4.34 ± 0.98 (4.37) | <0.0001 |
| Sodium [mmol/L] | 139.54 ± 34.61 (139.00) | 139.07 ± 14.98 (139.00) | 0.647 |
| Total cholesterol [mmol/l] | 4.72 ± 1.51 (4.54) | 4.09 ± 1.19 (4.00) | <0.0001 |
| Triglyceride [mmol/l] | 1.90 ± 1.79 (1.50) | 1.69 ± 0.96 (1.41) | 0.325 |
| White blood cell count [10^9^/L] | 8.67 ± 4.36 (7.89) | 10.25 ± 6.89 (8.86) | <0.0001 |

**Abbreviations:** BMI – body mass index; CRP – c-reactive protein; HbA1c – hemoglobin A1c; HCT – hematocrit, Hgb – hemoglobin, MCH – mean corpuscular hemoglobin, MCHC – mean corpuscular hemoglobin concentration, MCV – mean corpuscular volume, PDN – peripheral diabetic neuropathy. The mean value ± standard deviation (SD) with the median (in parentheses) was determined for each of the analyzed continuous parameters. In the case of binary parameters, we determined the sum of 1s and the percentage of all observations in the analyzed group. Individual comparisons of features between the groups with and without heart failure were performed by the Mann-Whitney U test (continuous) or the χ2 test (binary) according to data type.

**Supplementary Table 2**: Clinical patient parameters of Dataset B.

The most discriminative features for all patients are bold and red.

| **Parameter** | **Patients without Heart Failure (n=216)** | **Patients with Heart Failure**  **(n=49)** | ***p*-value** |
| --- | --- | --- | --- |
| ***Demographic parameters*** | | | |
| **Age** | **54.39 ± 17.36 (59.00)** | **70.59 ± 10.34 (70.00)** | **<0.0001** |
| Men, n (%) | 216 (54.63%) | 23 (46.94%) | 0.330 |
| ***Clinical parameters*** | | | |
| *Diabetes-related* | | | |
| BMI [kg/m^2^] | 29.15 ± 6.27 (28.35) | 34.25 ± 5.92 (33.60) | <0.0001 |
| Duration of diabetes [years] | 10.20 ± 10.31 (8.00) | 14.24 ± 9.19 (15.00) | 0.016 |
| **Type of diabetes [% of type 1]** | **58 (26.85%)** | **0 (0.00%)** | **<0.0001** |
| *Cardiovascular-related* | | | |
| **Atrial fibrillation** | **8 (3.76%)** | **9 (19.57%)** | **<0.001** |
| Carotid arteries stenosis | 4 (1.85%) | 2 (4.08%) | 0.343 |
| Coronary artery disease | 40 (18.52%) | 30 (61.22%) | <0.0001 |
| Hypertension | 138 (63.89%) | 47 (95.92%) | <0.0001 |
| Mean diastolic blood pressure [mmHg] | 78.07 ± 6.65 (78.00) | 77.40 ± 6.41 (79.00) | 0.936 |
| Mean heart rate [bpm] | 80.02 ± 13.31 (80.00) | 84.08 ± 14.06 (80.00) | 0.048 |
| Mean systolic blood pressure [mmHg] | 127.98 ± 12.99 (127.00) | 133.35 ± 16.44 (130.00) | 0.068 |
| Peripheral artery disease | 8 (3.70%) | 4 (8.16%) | 0.175 |
| Stroke | 16 (7.41%) | 4 (8.16%) | 0.856 |
| *Diabetic complications* | | | |
| Diabetic foot disease | 2 (0.93%) | 1 (2.04%) | 0.505 |
| Diabetic peripheral neuropathy | 17 (7.87%) | 4 (8.16%) | 0.945 |
| Retinopathy | 35 (16.20%) | 8 (16.33%) | 0.983 |
| *General* | | | |
| Current smoker [% of yes] | 41 (18.98%) | 13 (26.53%) | 0.236 |
| Emergency admission [% of yes] | 53 (24.54%) | 18 (36.73%) | 0.082 |
| Number of days of hospital stay | 7.15 ± 2.97 (7.00) | 8.45 ± 3.36 (8.00) | 0.004 |
| *Concomitant diseases* | | | |
| Degenerative disease of the spine | 100 (46.30%) | 30 (61.22%) | 0.059 |
| Hypercholesterolemia | 129 (59.72%) | 39 (79.59%) | 0.009 |
| Hypertriglyceridemia | 96 (44.44%) | 24 (48.98%) | 0.565 |
| **Hyperuricemia** | **81 (37.50%)** | **38 (77.55%)** | **<0.0001** |
| ***Laboratory parameters*** | | | |
| Alanine aminotransaminase [U/L] | 40.09 ± 54.15 (24.85) | 32.06 ± 35.08 (22.30) | 0.183 |
| Aspartate aminotransaminase [U/L] | 37.54 ± 65.43 (23.20) | 37.55 ± 40.02 (25.40) | 0.339 |
| Basophil count [10^9^/L] | 0.03 ± 0.03 (0.02) | 0.02 ± 0.01 (0.02) | 0.989 |
| CRP [mg/L] | 17.73 ± 51.17 (2.77) | 35.78 ± 68.69 (7.91) | <0.0001 |
| **eGFR [ml/min/1.73m^2^]** | **85.89 ± 31.10 (87.83)** | **61.03 ± 30.41 (53.15)** | **<0.0001** |
| Eosinophil count [10^9^/L] | 0.15 ± 0.14 (0.12) | 0.21 ± 0.32 (0.15) | 0.175 |
| HbA1c [%] | 9.50 ± 2.56 (9.07) | 8.87 ± 2.59 (8.50) | 0.124 |
| HCT [%] | 40.10 ± 5.40 (40.80) | 38.87 ± 4.81 (39.00) | 0.073 |
| Hgb [g/dL] | 13.68 ± 1.95 (14.00) | 13.05 ± 1.79 (13.20) | 0.015 |
| Ketones - urine sample | 58 (26.85%) | 5 (10.20%) | 0.013 |
| Lymphocyte count [10^9^/L] | 2.07 ± 0.98 (1.99) | 1.97 ± 0.87 (1.87) | 0.308 |
| MCH [pg] | 29.79 ± 2.30 (29.90) | 29.88 ± 2.32 (30.10) | 0.684 |
| MCHC [g/dL] | 34.10 ± 1.39 (34.20) | 33.52 ± 1.37 (33.70) | 0.004 |
| MCV [fL] | 87.34 ± 5.54 (86.90) | 89.05 ± 5.36 (89.20) | 0.015 |
| Mean fast. glycemia [mg/dL] first day | 224.22 ± 161.66 (184.00) | 206.51 ± 110.73 (177.30) | 0.606 |
| Mean fast. glycemia [mg/dL] last day | 132.57 ± 38.08 (127.00) | 142.80 ± 36.35 (137.00) | 0.043 |
| Mean post. glycemia [mg/dL] first day | 182.29 ± 75.50 (164.00) | 174.93 ± 55.66 (161.50) | 0.893 |
| Mean post. glycemia [mg/dL] last day | 141.34 ± 41.91 (135.00) | 147.28 ± 42.38 (145.00) | 0.160 |
| Monocyte count [10^9^/L] | 0.71 ± 0.71 (0.57) | 0.82 ± 0.36 (0.68) | 0.000 |
| Neutrophil count [10^9^/L] | 6.22 ± 5.20 (4.83) | 7.99 ± 4.37 (6.82) | <0.0001 |
| Platelet count [10^9^/L] | 260.42 ± 99.66 (249.00) | 253.02 ± 74.93 (247.00) | 0.808 |
| Potassium [mmol/L] | 4.60 ± 0.62 (4.58) | 4.48 ± 0.73 (4.54) | 0.478 |
| Protein - urine sample | 119 (55.09%) | 37 (75.51%) | 0.009 |
| Red blood cell count [10^12^/L] | 4.61 ± 0.66 (4.71) | 4.38 ± 0.58 (4.46) | 0.009 |
| Sodium [mmol/L] | 137.87 ± 5.18 (139.00) | 138.41 ± 4.71 (139.00) | 0.550 |
| Total cholesterol [mmol/l] | 4.62 ± 1.30 (4.42) | 4.23 ± 1.16 (3.87) | 0.075 |
| Triglyceride [mmol/l] | 1.93 ± 1.69 (1.50) | 1.84 ± 0.91 (1.59) | 0.487 |
| White blood cell count [10^9^/L] | 9.28 ± 6.37 (7.98) | 11.05 ± 4.49 (9.68) | <0.0001 |

**Abbreviations:** BMI – body mass index; CRP – c-reactive protein; HbA1c – hemoglobin A1c; HCT – hematocrit, Hgb – hemoglobin, MCH – mean corpuscular hemoglobin, MCHC – mean corpuscular hemoglobin concentration, MCV – mean corpuscular volume, PDN – peripheral diabetic neuropathy. The mean value ± standard deviation (SD) with the median (in parentheses) was determined for each of the analyzed continuous parameters. In the case of binary parameters, we determined the sum of 1s and the percentage of all observations in the analyzed group. Individual comparisons of features between the groups with and without heart failure were performed by the Mann-Whitney U test (continuous) or the χ2 test (binary) according to data type.

**Supplementary Table 3:** The values of the most discriminative features for Dataset B in individual scenarios (*i*) all patients: red; (*ii*) MASLD(+): blue; and (*iii*) MASLD(-) patients: yellow.

| **Parameter** | **(*i*) All: 265**  **(5 parameters)** | | | **(*ii*) MASLD(+): 137**  **(3 parameters)** | | | **(*iii*) MASLD(-): 128**  **(2 parameters)** | | |
| --- | --- | --- | --- | --- | --- | --- | --- | --- | --- |
|  | **Patients without HF (n=216)** | **Patients with HF (n=49)** | ***p*-value** | **Patients without HF (n=105)** | **Patients with HF (n=32)** | ***p*-value** | **Patients without HF (n=111)** | **Patients with HF (n=17)** | ***p*-value** |
| Age | 54.39 ± 17.36 (59.00) | 70.59 ± 10.34 (70.00) | <0.0001 | 58.83 ± 11.49 (60.00) | 67.88 ± 10.13 (69.00) | <0.001 | 50.19 ± 20.67 (50.00) | 75.71 ± 8.89 (74.00) | <0.0001 |
| Type of diabetes [% of type 1] | 58  (26.85%) | 0  (0.00%) | <0.0001 | 5  (4.78%) | 0  (0.00%) | 0.209 | 53 (47.75%) | 0  (0.00%) | <0.001 |
| Atrial fibrillation | 8  (3.76%) | 9 (19.57%) | <0.0001 | 5  (4.81%) | 5  (17.24%) | 0.039 | 3  (2.75%) | 4  (23.53%) | <0.0001 |
| Hyperuricemia | 81  (37.50%) | 38 (77.55%) | <0.0001 | 55 (52.38%) | 24 (75.00%) | 0.023 | 26  (23.42) | 14 (82.35%) | <0.0001 |
| eGFR [ml/min/1.73m^2^] | 85.89 ± 31.10 (87.83) | 61.03 ± 30.41 (53.15) | <0.0001 | 79.16 ± 25.94 (80.38) | 67.60 ± 32.38 (65.14) | 0.029 | 92.25 ± 32.21 (93.79) | 48.66 ± 22.23 (48.66) | <0.0001 |

The mean value ± standard deviation (SD) with the median (in parentheses) was determined for each of the analyzed continuous parameters. In the case of binary parameters, we determined the sum of 1s and the percentage of all observations in the analyzed group. Individual comparisons of features between the groups with and without heart failure were performed by the Mann-Whitney U test (for continuous parameters) or the χ2 test (for binary).

**Supplementary Table 4:** The ROC curve analysis for extracting the Model Operating Point using three methods (Index of Union, the Youden’s index and the closest to (0, 1) criteria) shows that all techniques lead to obtaining the machine learning models of similar classification performance.

| **Method** | *(i)* All (5 parameters) | | | *(i)* MASLD(+) subgroup (5 parameters) | | | *(ii)* MASLD(+) subgroup (3 parameters) | | | *(i)* MASLD(-) subgroup (5 parameters) | | | *(iii)* MASLD(-) subgroup (2 parameters) | | |
| --- | --- | --- | --- | --- | --- | --- | --- | --- | --- | --- | --- | --- | --- | --- | --- |
| **Cut off** | IoU | Distance | Youden | IoU | Distance | Youden | IoU | Distance | Youden | IoU | Distance | Youden | IoU | Distance | Youden |
| **Value** | 0.166 | 0.199 | 0.166 | 0.208 | 0.208 | 0.208 | 0.178 | 0.178 | 0.178 | 0.177 | 0.203 | 0.177 | 0.162 | 0.173 | 0.144 |
| Metric | | | | | | | | | | | | | | | |
| **True positive** | 256 | 236 | 256 | 131 | 131 | 131 | 134 | 134 | 134 | 103 | 99 | 103 | 103 | 101 | 109 |
| **False positive** | 423 | 341 | 423 | 163 | 163 | 163 | 202 | 202 | 202 | 161 | 142 | 161 | 184 | 176 | 200 |
| **False negative** | 61 | 81 | 61 | 63 | 63 | 63 | 60 | 60 | 60 | 20 | 24 | 20 | 20 | 22 | 14 |
| **True negative** | 995 | 1077 | 995 | 634 | 634 | 634 | 595 | 595 | 595 | 460 | 479 | 460 | 437 | 445 | 421 |
| **Sensitivity** | 0.808 | 0.744 | 0.808 | 0.675 | 0.675 | 0.675 | 0.691 | 0.691 | 0.691 | 0.837 | 0.805 | 0.837 | 0.837 | 0.821 | 0.886 |
| **Specificity** | 0.702 | 0.760 | 0.702 | 0.795 | 0.795 | 0.795 | 0.747 | 0.747 | 0.747 | 0.741 | 0.771 | 0.741 | 0.704 | 0.717 | 0.678 |
| **AUC** | 0.836 | 0.836 | 0.836 | 0.816 | 0.816 | 0.816 | 0.781 | 0.781 | 0.781 | 0.865 | 0.865 | 0.865 | 0.843 | 0.843 | 0.843 |
| **AUC CI1** | 0.816 | 0.816 | 0.816 | 0.787 | 0.787 | 0.787 | 0.750 | 0.750 | 0.750 | 0.836 | 0.836 | 0.836 | 0.812 | 0.812 | 0.812 |
| **AUC CI2** | 0.856 | 0.856 | 0.856 | 0.846 | 0.846 | 0.846 | 0.811 | 0.811 | 0.811 | 0.893 | 0.893 | 0.893 | 0.873 | 0.873 | 0.873 |
| **CC with event [%]** | 80.757 | 74.448 | 80.757 | 67.526 | 67.526 | 67.526 | 69.072 | 69.072 | 69.072 | 83.740 | 80.488 | 83.740 | 83.740 | 82.114 | 88.618 |
| **CC without event [%]** | 70.169 | 75.952 | 70.169 | 79.548 | 79.548 | 79.548 | 74.655 | 74.655 | 74.655 | 74.074 | 77.134 | 74.074 | 70.370 | 71.659 | 67.794 |
| **CC All [%]** | 72.104 | 75.677 | 72.104 | 77.195 | 77.195 | 77.195 | 73.562 | 73.562 | 73.562 | 75.672 | 77.688 | 75.672 | 72.581 | 73.387 | 71.237 |

**Supplementary Table 5:** Results of predicting the occurrence of HF using three MLR models based on the most discriminative features, extracted for (*i*) all patients, (*ii*) MASLD(+) patients, and (*iii*) MASLD(-) patients from Dataset B.

The best results in the subgroup analysis are boldfaced.

| **Method** | **Sensitivity** | **Specificity** | **CC with event [%]** | **CC without event [%]** | **CC All [%]** |
| --- | --- | --- | --- | --- | --- |
| *(i)* All (5 parameters) | 0.80 | 0.71 | 79.59 | 70.83 | 72.45 |
| *(i)* MASLD(+) subgroup (5 parameters) | **0.66** | **0.74** | **65.63** | **74.29** | **72.26** |
| *(ii)* MASLD(+) subgroup (3 parameters) | 0.63 | 0.66 | 62.50 | 65.71 | 64.96 |
| *(i)* MASLD(-) subgroup (5 parameters) | **0.82** | **0.79** | **82.35** | **79.28** | **79.69** |
| *(iii)* MASLD(-) subgroup (2 parameters) | **0.82** | 0.78 | **82.35** | 78.38 | 78.91 |

**Supplementary Table 6**: Pharmacological patient record of Dataset A.

| Parameter | Patients without Heart Failure (n=1418) | Patients with Heart Failure  (n=317) | p-value |
| --- | --- | --- | --- |
| Pharmacotherapy | | | |
| ACEi/ARB | 756 (53.31%) | 155 (48.90%) | 0.154 |
| Allopurinol | 255 (17.98%) | 131 (41.32%) | <0.0001 |
| Alpha blocker | 132 (9.31%) | 52 (16.40%) | <0.001 |
| Amiodarone | 3 (0.21%) | 7 (2.21%) | <0.0001 |
| ASA | 656 (46.26%) | 195 (61.51%) | <0.0001 |
| Beta blocker | 643 (45.35%) | 268 (84.54%) | <0.0001 |
| Calcium blocker | 375 (26.45%) | 98 (30.91%) | 0.106 |
| Clopidogrel | 45 (3.17%) | 36 (11.36%) | <0.0001 |
| Digoxin | 5 (0.35%) | 23 (7.26%) | <0.0001 |
| DPP-4 inhibitors | 219 (15.44%) | 54 (17.03%) | 0.482 |
| Fibrate | 26 (1.83%) | 6 (1.89%) | 0.944 |
| GLP-1 agonist | 31 (2.19%) | 4 (1.26%) | 0.290 |
| Heparin | 50 (3.53%) | 44 (13.88%) | <0.0001 |
| Insulin | 1158 (81.66%) | 208 (65.62%) | <0.0001 |
| PPI | 337 (23.77%) | 178 (56.15%) | <0.0001 |
| Loop diuretic | 273 (19.25%) | 242 (76.34%) | <0.0001 |
| Metformin | 718 (50.63%) | 122 (38.49%) | <0.0001 |
| NOAC | 48 (3.39%) | 57 (17.98%) | <0.0001 |
| Non-loop diuretics | 248 (17.49%) | 25 (7.89%) | <0.0001 |
| Potassium-sparing diuretics | 63 (4.44%) | 110 (34.70%) | <0.0001 |
| SGLT-2 inhibitor | 183 (12.91%) | 20 (6.31%) | 0.001 |
| Statin | 727 (51.27%) | 183 (57.73%) | 0.037 |
| Sulfonylureas | 359 (25.32%) | 114 (35.96%) | <0.001 |
| VKA | 18 (1.27%) | 35 (11.04%) | <0.0001 |

**Abbreviations:**

The sum of 1s and the percentage of all observations in the analyzed group are determined. Individual comparisons of features between the groups with and without heart failure were performed by = the χ2 test.

**Supplementary Table 7**: Pharmacological patient record of Dataset B.

| Parameter | Patients without Heart Failure (n=1418) | Patients with Heart Failure (n=317) | p-value |
| --- | --- | --- | --- |
| Pharmacotherapy | | | |
| ACEi/ARB | 109  (50.46%) | 29 (59.18%) | 0.270 |
| Allopurinol | 53  (24.54%) | 30 (61.22%) | <0.0001 |
| Alpha blocker | 16  (7.41%) | 10 (20.41%) | 0.006 |
| Amiodarone | 0    (0.00%) | 4 (8.16  %) | <0.0001 |
| ASA | 71  (32.87%) | 21 (42.86%) | 0.185 |
| Beta blocker | 103  (47.69%) | 45 (91.84%) | <0.0001 |
| Calcium blocker | 52  (24.07%) | 18 (36.73%) | 0.070 |
| Clopidogrel | 5    (2.31%) | 5 (10.20%) | 0.009 |
| Digoxin | 5    (2.31%) | 3 (6.12  %) | 0.160 |
| DPP-4 inhibitors | 11  (5.09%) | 2 (4.08  %) | 0.767 |
| Fibrate | 2    (0.93%) | 1 (2.04  %) | 0.505 |
| GLP-1 agonist | 6    (2.78%) | 2 (4.08  %) | 0.630 |
| Heparin | 9    (4.17%) | 7 (14.29%) | 0.007 |
| Insulin | 152  (70.37%) | 34 (69.39%) | 0.892 |
| PPI | 48  (22.22%) | 27 (55.10%) | <0.0001 |
| Loop diuretic | 31  (14.35%) | 35 (71.43%) | <0.0001 |
| Metformin | 113  (52.31%) | 18 (36.73%) | 0.049 |
| NOAC | 13  (6.02%) | 13 (26.53%) | <0.0001 |
| Non-loop diuretics | 28  (12.96%) | 5 (10.20%) | 0.597 |
| Potassium-sparing diuretics | 10  (4.63%) | 22 (44.90%) | <0.0001 |
| SGLT-2 inhibitor | 48  (22.22%) | 10 (20.41%) | 0.782 |
| Statin | 86  (39.81%) | 35 (71.43%) | 0.000 |
| Sulfonylureas | 28  (12.96%) | 8 (16.33%) | 0.535 |
| VKA | 3    (1.39%) | 4 (8.16  %) | 0.008 |

**Abbreviations:**

The sum of 1s and the percentage of all observations in the analyzed group are determined. Individual comparisons of features between the groups with and without heart failure were performed by = the χ2 test.
